# Carcinoma In Situ (CIS): Is There a Difference in Efficacy between Various BCG Strains? A Comprehensive Review of the Literature

**DOI:** 10.3390/cancers16020245

**Published:** 2024-01-05

**Authors:** Andres Llano, Amy Chan, Cynthia Kuk, Wassim Kassouf, Alexandre R. Zlotta

**Affiliations:** 1 Division of Urology, Department of Surgical Oncology, Department of Surgery, Sinai Health System, University of Toronto, Toronto, ON M5G 2N2, Canada; andres.llano@mail.utoronto.ca (A.L.);; 2 Division of Urology, McGill University Health Center, Montreal, QU H4A 3J1, Canada; wassim.kassouf.med@ssss.gouv.qc.ca; 3 Division of Urology, Department of Surgical Oncology, Department of Surgery, Princess Margaret Cancer Centre, University Heath Network, University of Toronto, Toronto, ON M5G 2C4, Canada

**Keywords:** bladder cancer, Bacillus Calmette-Guérin, Carcinoma in situ, BCG, CIS

## Abstract

**Simple Summary:**

Carcinoma in situ of the bladder is an aggressive type of non-muscle invasive bladder cancer characterized as a flat, high-grade tumour confined to the urothelial layer. Non-muscle invasive bladder cancer comprises approximately 75% to 80% of all bladder cancers, with Carcinoma in situ found in about 10% of cases. Intravesical instillations of Bacillus Calmette-Guérin immunotherapy is the standard of care for high-risk and intermediate-risk papillary non-muscle invasive bladder cancer as well as for Carcinoma in situ. Evidence supports that the different Bacillus Calmette-Guérin strains, despite genetic variability, are equally effective clinically for preventing the recurrence and progression of papillary non-muscle invasive bladder cancer. The available evidence regarding possible differences in clinical efficacy between various Bacillus Calmette-Guérin strains specifically against Carcinoma in situ is lacking. We therefore reviewed the literature on this topic. We found that the clinical efficacy of the various Bacillus Calmette-Guérin strains against Carcinoma in situ appears similar. However, our conclusions should be considered with caution as most studies were underpowered, and none of the trials were designed as head-to-head comparisons. Randomized studies should be encouraged in this space to draw definitive conclusions.

**Abstract:**

**Introduction:** Intravesical Bacillus Calmette-Guérin (BCG) immunotherapy is the standard of care for high-risk and intermediate-risk non-muscle-invasive bladder cancer (NMIBC) as well as for Carcinoma in situ (CIS). Evidence supports that the different BCG strains, despite genetic variability, are equally effective clinically for preventing the recurrence and progression of papillary NMIBC. The available evidence regarding possible differences in clinical efficacy between various BCG strains in CIS is lacking. **Methods:** We reviewed the literature on the efficacy of different BCG strains in patients with CIS (whether primary, secondary, concomitant, or unifocal/multifocal), including randomized clinical trials (RCTs), phase II/prospective trials, and retrospective studies with complete response rates (CRR), recurrence-free survival (RFS), or progression-free survival (PFS) as endpoints. **Results:** In most studies, being RCTs, phase II prospective trials, or retrospective studies, genetic differences between BCG strains did not translate into meaningful differences in clinical efficacy against CIS, regardless of the CIS subset (primary, secondary, or concurrent) or CIS focality (unifocal or multifocal). CRR, RFS, and PFS were not statistically different between various BCG strains. None of these trials were designed as head-to-head comparisons between BCG strains focusing specifically on CIS. Limitations include the small sample size of many studies and most comparisons between strains being indirect rather than head-to-head. **Conclusions:** This review suggests that the clinical efficacy of the various BCG strains appears similar, irrespective of CIS characteristics. However, based on the weak level of evidence available and underpowered studies, randomized studies in this space should be encouraged as no definitive conclusion can be drawn at this stage.

## 1. Introduction

Carcinoma in situ (CIS) of the bladder is an aggressive type of non-muscle invasive bladder cancer (NMIBC) characterized as a flat, high-grade tumour confined to the urothelial layer. NMIBC comprises approximately 75% to 80% of all bladder cancers, with about 10% of these cases having CIS [1,2]. An important distinction of bladder CIS compared to CIS diseases affecting other organs is that it is not a precursor of malignancy but rather an aggressive malignant entity by itself [2].

In papillary NMIBC, the presence of CIS increases the risk of recurrence and progression to invasive disease and metastatic spread [2,3,4]. CIS is a key prognostic factor of NMIBC and an early independent predictor of time to first recurrence and of progression to more invasive forms of the disease [5,6]. CIS can be further stratified into primary, secondary, and concomitant/concurrent CIS [7]. Primary CIS is typically defined as CIS without a previous or concurrent papillary tumour, and secondary CIS as lesions detected during the follow-up of patients with a previous diagnosis of papillary tumour(s) [8]. Patients presenting with primary CIS seem to have worse oncological outcomes compared to those with secondary CIS [9]. Concomitant CIS is defined as CIS detected simultaneously with non-muscle invasive papillary tumours. It is unclear whether difference in oncological outcome exist between primary and secondary CIS. 

CIS is also often described according to its distribution pattern, either unifocal (one positive biopsy core) or multifocal (two or more positive cores) [7,9]. Most of the literature in the field distinguishing between unifocal and multifocal CIS was published before the use of new imaging modalities like blue light [10,11,12,13]. 

The tumour–node–metastasis (TNM) bladder cancer staging system categorizes pure CIS (also called Tis, which is CIS without associated papillary tumours) as a separate entity from papillary tumours (Ta, confined to the surface, and T1, invading into the lamina propria without invasion into the muscle) [14]. Pure CIS has a distinct molecular profile compared to NMIBC [15]. New developments in imaging technology, including Photodynamic Detection (PDD) and Narrow Band Imaging (NBI), have markedly enhanced the detection of CIS and possibly reduced recurrence rates [10,11,12,13]. However, one recent randomized study did not find that PDD reduced recurrence rates, nor was it more cost-effective compared to white light cystoscopy in the setting of the routine post-operative instillation of intravesical chemotherapy [16]. 

In current practice, the standard treatment for CIS, as well as intermediate- and high-risk papillary NMIBC [1], includes a transurethral resection of bladder tumour (TURBT) followed by intravesical Bacillus Calmette-Guérin (BCG) immunotherapy to prevent the recurrence and progression of the disease [17]. BCG is a non-virulent mycobacterium isolated from *Mycobacterium bovis*, a mycobacterial strain close to *M. tuberculosis* but which affects cattle, by Calmette and Guérin at the Pasteur Institute of Paris [17]. It took over 200 passages and 13 years from 1908 to 1921 to attenuate the virulent strain to a weakened form which could be used as a vaccine [18]. Several strains were developed from the initial French strain and distributed worldwide. BCG evolved differently in each laboratory throughout the world due to various protocols for maintenance and production, resulting in different substrains. BCG’s mode of action in NMIBC is still not fully known but is primarily believed to mediate immunity through the development of cellular immunity and possibly also trained immunity [18].

**Figure 1:**
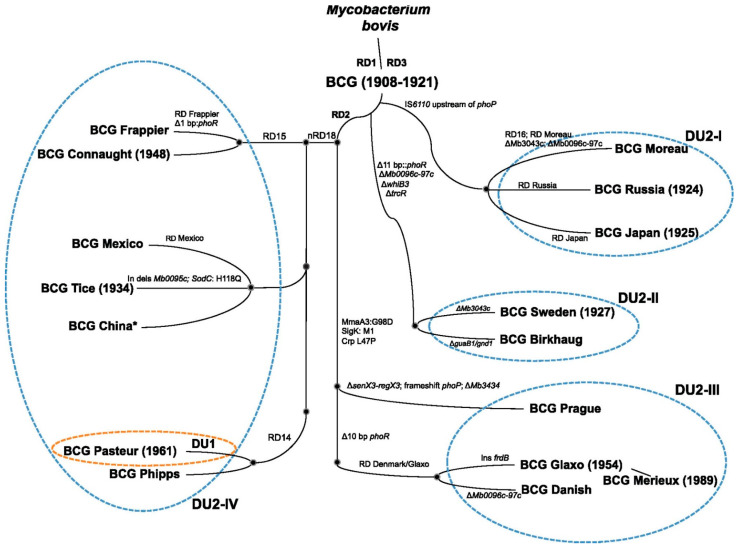
Evolutionary timeline of *Mycobacterium bovis.* The brown and blue dashed ellipses indicate tandem duplications DU1 and DU2, which enable classification of BCG strains into four major lineages. * BCG China/BCG Beijing belongs to a cluster closely related to BCG Danish. Reproduced with permission from Bottai, Daria, and Roland Brosch. “The BCG Strain Pool: Diversity Matters” [19].

The different BCG strains currently available vary based on their genomic variations during their evolutionary history: either early or late (Figure 1) [19]. The loss of RD1, the major difference between *M. bovis* and BCG, is present in all subsequent cultured strains [19]. As genetic alterations in a strain lead to differences in antigenicity and clinical characteristics [17,18], a key question is whether the BCG strains differ in their clinical efficacy [20]. While one randomized study (which did not use maintenance regimens) argues that there are indeed differences between strains [21], the vast majority of other studies suggest that all BCG strains are considered equally effective treatments for NMIBC with similar toxicity profiles, although variations have been described [20,22,23,24,25,26,27,28]. 

One explanation stems from the mechanism of action of BCG. Because so many components of the live attenuated mycobacteria, irrespective of the strain, are able to activate the robust immune cascade that ultimately results in tumour-cell killing, it is unlikely that variabilities between strains play a major role [27,29]. However, not only the characteristics of mycobacteria play a role but also treatment regimens, the concentration used, gender, age, and race, among others, as well as a combination of human and mycobacteria factors make interpretation and drawing conclusions difficult.

Although BCG is the mainstay in CIS, surprisingly, the variability in clinical efficacy of the BCG strains has not been specifically studied in this condition. Previous studies have treated CIS as a dichotomous variable [6], though some studies found that primary, secondary, and concomitant CIS harbour a different underlying biology [30]. The goal of this review is to analyze the available evidence regarding the clinical efficacy of various BCG strains specifically against CIS.

## 2. Evidence Synthesis

We searched PubMed, Scopus, and Embase for articles using different BCG strains for the treatment of NMIBC with CIS and pure CIS from 1980 to September 2023. We used the terms “Carcinoma in situ”, “CIS”, “unifocal”, “multifocal”, “concurrent”, “primary”, “secondary”, “non-muscle invasive”, “bladder cancer”, “urothelial carcinoma”, “superficial”, “randomized”, “retrospective”, “prospective”, “recurrence”, “progression”, “outcomes”, “mortality”, “BCG”, “bacillus Calmette Guerin”, “strain”, and “genetics”. None of the studies that focused on CIS was designed as head-to-head comparisons between BCG strains. Table 1, Table 2, Table 3 and Table 4 summarize the available evidence we gathered regarding BCG efficacy for the treatment of primary, secondary, and concurrent CIS in available randomized controlled trials (RCTs), phase II/prospective trials, and retrospective studies. 

## 3. Randomized Controlled Trials 

Table 1 details the available RCT studies that investigated the use of different BCG strains in patients with CIS [31,32,33,34,35,36,41]. As previously mentioned, CIS was not the primary endpoint in any of these randomized studies, as they included papillary tumours. The complete response rates varied from 50.7% to 86.5% [36,41]. In a study that compared BCG Connaught monotherapy to alternating BCG/mitomycin C (MMC) chemotherapy in patients with CIS (*n* = 304) [31], the risk of recurrence was significantly lower in the BCG group compared to the MMC/BCG group (49% vs. 59% at 15 years, *p* = 0.048), but patients demonstrated comparable rates of progression, overall survival (OS), and disease-specific mortality. Disease recurrence was higher in patients with primary CIS compared to those with concomitant CIS (70% vs. 34%, respectively, *p* = 0.055). Yokomizo et al. [32] compared a standard dosage (80 mg) to a half dosage (40 mg) of BCG Tokyo in patients with primary (*n* = 65) and concomitant (*n* = 80) CIS. They did not find significant differences in the complete response rate (CRR), recurrence, progression, or OS. In a study which compared BCG Connaught to epirubicin chemotherapy in 168 patients with CIS, with 52% having concurrent CIS [34], no significant differences in CRR were observed (overall CRR of 56% with epirubicin vs. 65% with BCG, *p* = 0.21). However, the time to recurrence was significantly longer in patients treated with BCG after having achieved a CR, with CIS recurrences more frequently observed in complete responders to epirubicin (45% vs. 16%). An RCT [35] evaluated the effects of reducing the BCG Connaught dosage in T1G3 and CIS patients with no difference observed in patients with primary (*n* = 23) and concomitant CIS (*n* = 42).

Finally, one randomized single-institution study trial compared BCG Connaught and Tice for the treatment of 142 high-risk NMIBC patients [21]. This study had an important imbalance in the number of patients included in each arm (71 treated with BCG Connaught, 60 treated with Tice) but similar percentages of CIS (44% for patients treated with BCG Connaught, and 43% CIS for those treated with Tice). Three patients (4%) treated with BCG Connaught and nine patients (15%) treated with Tice presented with Tis. Whereas the 5-year recurrence-free survival (RFS) of patients treated with BCG Connaught (74.0%; 95% CI, 62.8–87.2) was significantly greater than that of those treated with BCG Tice (48.0%; 95% CI, 35.5–65.1; *p* = 0.0108). The 5-year progression-free survival (PFS) was quite high in both arms and not significantly different (94.1%, 95% CI87.8–100 BCG Connaught vs. 87.9%, 95% CI 76.5–100 BCG Tice, *p* = 0.3442). A majority limitation of this study was that no maintenance regimens were administered in any of the study arms, though this study was initiated before BCG maintenance was shown to be beneficial. 

## 4. Phase II Trials and Prospective Studies

In two phase II BCG clinical trials (Table 2), Jakse and colleagues [37] and deKernion and colleagues [38] examined the efficacy of BCG Connaught and TICE, respectively, on patients with CIS of any type. BCG Connaught achieved a 75% CRR in 103 CIS patients, while BCG TICE had a 68% CRR in 19 CIS patients. Rosevear et al. [39] analyzed the outcome in patients with pure CIS (*n* = 146) and papillary disease + CIS (*n* = 85) treated with a combination of BCG TICE or Connaught with interferon-α therapy. Comparable CCRs were observed. 

Table 3 summarizes the evidence regarding prospective studies [21,24,40,42,43]. A prospective comparison between BCG Tokyo and Connaught strains [24] found no significant difference between strains in terms of CRR or RFS at 5 years. In a sub-analysis of Ta/T1 patients without CIS (*n* = 78, 35 for the Tokyo strain and 43 for the Connaught strain), no statistically significant difference in the 5-year RFS between these groups were observed either. An analysis conducted by Gofrit and colleagues [40] that compared primary CIS patients (*n* = 38) with concomitant CIS patients (*n* = 66) using BCG Connaught demonstrated similar outcomes for RFS, PFS, OS, and cancer-specific survival (CSS) between groups. Takenaka and colleagues [41] using BCG Tokyo did not observe significant differences in CRR, RFS, or PFS for primary CIS (*n* = 62), secondary CIS (*n* = 63), or concomitant CIS (*n* = 60). These authors further subdivided the patient based on CIS extension: 75 patients (40.5%) had limited CIS (defined as less than three positive sites out of four to six biopsy sites), and 64 patients (34.6%) had extensive CIS (more than three positive sites out of four to six biopsies). CIS extension was an independent prognostic factor for progression (*p* = 0.02). In another prospective analysis including 43 CIS patients (13 with primary CIS and 30 with secondary or concomitant CIS) who received BCG Tokyo [42], no difference was observed between groups with respect to CRR or RFS at 5 years. The median CR duration was 39 and 36 months in the primary CIS group and secondary/concomitant CIS group, respectively. Likewise, there was no significant difference when patients were subdivided into focal and diffuse CIS extensions. Finally, a prospective trial by Sood et al. [43] compared 80 and 120 mg doses of intravesical BCG (Moscow I, Russian strain manufactured by Serum Institute of India) in patients with intermediate- and high-risk NMIBC with CIS (*n* = 51) and without CIS (*n* = 53). No statistically significant difference between groups was observed, although the sample size was again fairly small.

## 5. Retrospective Studies

Retrospective studies are detailed and summarized in Table 4 [7,44,54]. It is important to note that none of these retrospective studies were primarily designed to compare various BCG strains in patients with CIS. For instance, Ferro and colleagues [44] assessed the impact of age on survival outcomes in these patients. A comparative study by Del Giudice and colleagues [45] assessed differences between three BCG strains (Connaught, TICE, and RIVM) in patients with Ta, T1, and CIS tumours. The 5-year RFS and PFS were longest for TICE (61.3% and 81%, respectively) compared to RIVM (60.2% and 78.2%) and Connaught (54.1% and 74.7%). When adjusting for risk factors such as age, gender, tumour number, prior recurrence, T category, tumour grade, and the presence of CIS, a statistically significant difference was found for the time to first recurrence for both TICE and RIVM compared to Connaught (TICE = RIVM > Connaught). However, a head-to-head comparison in treated CIS patients was not reported, and whether the different strains used or unmeasured confounders played a role remains unclear. Another retrospective study [46] looked at differences in response to BCG for primary (*n* = 98) and concomitant (*n* = 51) CIS patients. PFS, but not CSS or OS, was significantly different between these two CIS groups (86% and 71%, respectively, *p* = 0.03). The efficacy of BCG Pasteur was compared in primary (*n* = 13), concomitant (*n* = 28), and secondary (*n* = 6) CIS patients [47]. No significant differences between CIS groups for CRR, 5-year PFS, and OS, were found, though the sample size was very small. 

In one of the largest studies to date on T1G3 patients treated with BCG, Witjes and colleagues [48] compared the efficacy of BCG TICE and Connaught strains in 2,099 patients (*n* = 1572 without CIS, *n* = 527concomitant CIS). The authors conducted an additional analysis to understand the effect of different treatment schedules on survival outcomes. The time to first recurrence favoured Connaught (*p* = 0.047), but there was no difference between strains in the time to progression, even after adjusting for the presence of CIS. Moreover, there was no significant difference between strains in survival or the time to death due to bladder cancer. In another study that evaluated exclusively primary CIS patients (*n* = 155) treated with BCG Tokyo [9], no association between CIS pattern (i.e., unifocal, multifocal, or diffuse) and BCG response, disease recurrence, or progression was observed. BCG Tokyo’s efficacy was evaluated in another retrospective study [49] and as shown in other studies, the presence of concomitant CIS was an independent risk factor of disease progression and survival. BCG TICE and Moreau were compared in 660 patients with NMIBC (*n* = 15 Tis and *n* = 88 concomitant CIS) [50], and no significant difference in RFS or PFS were observed. Another comparative study [51] looked at BCG TICE versus BCG RIVM using a 1:1 propensity-matched NMIBC study population including 133 patients treated with TICE (*n* = 15 concomitant CIS and *n* = 118 without CIS) and 133 matched patients who received RIVM (*n* = 10 with concomitant CIS and *n* = 123 without CIS). No significant differences between strains were found after 5 years for RFS (56% TICE vs. 48% RIVM), PFS (77% TICE vs. 79% RIVM), or CSS (96.2% TICE vs. 90.7% RIVM). BCG TICE was associated with a longer time to first recurrence compared to BCG RIVM. 

Nowak and colleagues [52] conducted a three-arm investigation with Moreau, TICE, and RIVM strains in 590 patients with T1HG tumours (*n* = 119 with concomitant CIS, *n* = 466 without CIS, and *n* = 5 unknown). No significant differences were observed in the 5-year RFS (70.5% Moreau vs. 66.7% TICE vs. 55.2% RIVM) or PFS (84% Moreau vs. 85% TICE vs. 77.8% RIVM) between strains.

Koguchi and colleagues [53] examined the efficacy of BCG Tokyo maintenance versus induction therapy in 60 Ta/T1 patients and in a small sample of 18 Tis patients. The maintenance regimen was superior to induction alone regarding RFS (89.5% vs. 65.0%, respectively, *p* = 0.02). In contrast, Griffiths and colleagues [54] investigated the efficacy of an unspecified BCG strain in 23 primary CIS patients, 37 Ta + CIS patients, and 75 T1 + CIS patients. The authors reported comparable CRRs at 3 months between the three groups (74% primary CIS vs. 70% CIS + Ta vs. 75% CIS + T1). T1 + CIS patients demonstrated worse outcomes for 5-year PFS and CSS (PFS: 80% primary CIS vs. 82% CIS + Ta vs. 51% CIS + T1, *p* = 0.013; CSS: 83% primary CIS vs. 86% CIS + Ta vs. 59% CIS + T1, *p* = 0.081).

## 6. Conclusions

This review summarized the currently available evidence comparing BCG strain efficacy on CIS. With some exceptions, it confirms what was observed for papillary NMIBC, namely that molecular differences between BCG strains did not translate into meaningful clinical differences in clinical efficacy in patients with CIS. Based on the indirect evidence presented in the present review, it seems that the various BCG strains likely have a similar efficacy against CIS, regardless of CIS subset, whether primary, secondary, concomitant, unifocal or multifocal. These conclusions should be tempered and be taken with caution. Studies included in this review predominantly assessed two particular strains of BCG (Connaught and Tokyo), and most studies included a small number of CIS patients, making these comparisons underpowered. Furthermore, none of these trials were designed as head-to-head comparisons between BCG strains focusing specifically on CIS as the primary endpoint. The inherent heterogeneity of CIS makes such comparisons even more complex and challenging. Pure CIS is relatively uncommon, but studies comparing different BCG strains in this specific subset should be encouraged to provide more robust and definitive answers. The S1602 Intergroup trial is a randomized phase III non-inferiority clinical trial that aims to compare the Tokyo-172 BCG strain to TICE BCG in BCG naïve high-grade NMIBC. Patients with pure CIS, Ta, T1, and Ta/T1 + CIS have been recruited for this trial, and robust comparisons between strains will be possible, albeit with the caveat of subset analyses. This trial may shed some light on this topic, although its primary outcome does not focus specifically on CIS [55]. Because BCG therapy, even in CIS, may not provide 100% efficacy in all patients, many therapeutic strategies are now being explored in pre-clinical and ongoing clinical trials to overcome BCG resistance. These include the combination of BCG with various immune checkpoint inhibitors in patients who are BCG refractory (reviewed by Chu & Pietzak, 2023) [56].

## Figures and Tables

**Table 1:** Randomized controlled trials (RCTs) using BCG for CIS.

Study	Strain	Schedule	Median Follow-Up	Number of CIS Patients	CRR	RFS	PFS	OS
Rentsch et al. (2014) [21]	Connaught vs. Tice	6 weeks vs. 6 weeks	47.6 months	CIS (any type): 31 vs. 26	NA	74% vs. 48%, *p* = 0.011	94.1% vs. 87.9%, *p* = 0.344	84.9% vs. 93.6%, *p* = 0.265
Kaasinen et al. (2016) [31]	Connaught	BCG 6 weeks + 10 months vs. MMC 6 weeks + BCG/MMC, 10 months alternating	119 months	Primary CIS: 91 Secondary CIS: 129 Concomitant CIS: 84	NA	26% reduction in risk of recurrence (BCG group). Risk of recurrence was significantly higher in primary CIS vs. concomitant CIS (HR = 0.66, *p* = 0.043)	No difference (BCG vs. MMC/BCG). Disease progression most likely in concomitant CIS, least likely in primary CIS (HR = 0.57, *p* = 0.055)	No difference (BCG vs. MMC/BCG)
Yokomizo et al. (2016) [32]	Tokyo	8 weeks SD vs. 1/2D	NA	Pure CIS: 65 Ta + CIS: 46 T1 + CIS: 34	85% SD vs. 78% 1/2D (no difference between CIS groups)	No difference	No difference	No difference
Koga et al. (2010) [33]	Tokyo	8 weeks vs. 8 weeks + 4 months Maintenance for 8 weeks	28.7 months	CIS (any type): 46	77%	74.1% (8 week) vs. 95.8% (M) at 2 years	No difference	92.6% vs. 97.1% (M)
de Reijke et al. (2005) [34]	Connaught	BCG (6 weeks + 3 weeks/3 years) vs. epirubicin (8 weeks + 3 weeks/3 years)	67 months	Primary CIS: 39 Secondary CIS: 41 Concurrent CIS: 88	63% vs. 60% (epi vs. BCG) 59% vs. 63% (epi vs. BCG) 51% vs. 69% (epi vs. BCG)	NA	NA	NA
Martínez-Piñeiro et al. (2005) [35]	Connaught	BCG (6 weeks + Q2W × 6) SD vs. 1/3D	61 months	Primary CIS: 23 Associated CIS: 42	NA	61.9% (SD) vs. 49.8% (1/3D) at 5 years (no difference between CIS groups)	74.3% (SD) vs. 73.5% (1/3D) at 5 years (no difference between CIS groups)	NA
Lamm et al. (2000) [36]	Connaught	6 weeks vs. 6 weeks + (M) (3 weeks/3 years)	Until death	CIS (any type): 278	68.1% (6 week) vs. 83.8% (M)	41% (6 weeks) vs. 60% (M) at 5 years	70% (6 weeks) vs. 76% (M) at 5 years	78% vs. 83% (M)

BCG = Bacillus Calmette-Guérin; CIS = Carcinoma in situ; CRR = complete remission rate; D = dose; HR = hazard ratio; MMC = mitomycin C; NA = not available; OS = overall survival; PFS = progression-free survival; RCT = randomized controlled trial; RFS = recurrence-free survival; SD = standard dose; (M) = maintenance.

**Table 2:** Phase II clinical trials using BCG for CIS.

Study	Design	Strain	Schedule	Median Follow-Up	Number of CIS Patients	CRR	RFS	PFS	OS
Jakse et al. (2001) [37]	Phase II trial	Connaught	6 weeks (+6 weeks if no CR)	91.2 months	CIS (any type): 103	75%	NA	NA	NA
deKernion et al. (1985) [38]	Phase II trial	TICE	8 weeks + 12 months	NA	CIS (any type): 19	68%	NA	NA	NA
Rosevear et al. (2011) [39]	Phase II trial	TICE or Connaught + Interferon-α	6 weeks + 3 weeks/15 months BCG mixed with 50 MU IFN-α-2b	24 months	Pure CIS: 146 Papillary + CIS: 85 (CIS + Ta: 45; CIS + T1: 36; CIS + Ta + T1: 4)	No difference in pure CIS vs. papillary + CIS groups *	NA	NA	NA

* Applies only to a patient’s current tumour staging. Patients who had a prior tumour diagnosed as papillary had a greater complete response rate than those with a prior tumour diagnosed as papillary + CIS. BCG = Bacillus Calmette-Guérin; CIS = Carcinoma in situ; CR = complete remission; CRR = complete remission rate; NA = not available; OS = overall survival; PFS = progression-free survival; RFS = recurrence-free survival.

**Table 3:** Prospective studies using BCG for CIS.

Study	Strain	Schedule	Median Follow-Up	Number of CIS Patients	CRR	RFS	PFS	OS
Sengiku et al. (2013) [23]	Tokyo vs. Connaught	Single course (6–8 doses)	855 days	pTis: 14; Papillary + CIS: 17 pTis: 15; Papillary + CIS: 5	90.3% 85%	61.8% at 5 years 56% at 5 years	NA	NA
Gofrit et al. (2009) [40]	Connaught	6 weeks induction, 46% received maintenance	75 months	Pure CIS: 38 Concomitant CIS: 66	NA	63% and 54% at 5 and 10 years (no difference between CIS groups)	79% and 77% at 5 and 10 years (no difference between CIS groups)	56.7% (no difference between CIS groups)
Takenaka et al. (2008) [41]	Tokyo	8 weeks	37.5 months	Primary CIS: 62 Secondary CIS: 63 Concomitant CIS: 60	86.5% (no difference between CIS groups)	66% at 5 years (no difference between CIS groups)	78.5% at 5 years (no difference between CIS groups)	NA
Mugiya et al. (2005) [42]	Tokyo	BCG 1/2D 6 weeks	54 months	Primary CIS: 13 Secondary CIS: 30	85% 83%	61.9% at 5 years (no difference between CIS groups)	NA	NA
Sood et al. (2020) [43]	Moscow, Russian I (80 mg vs. 120 mg)	6 weeks + 3 weeks/3 years	36 months	Intermediate- and high-risk NMIBC with or without CIS: 51 (80 mg) vs. 53 (120 mg)	NA	84.31% (80 mg) vs. 86.79% (120 mg) at 3 years	84.31% (80 mg) vs. 94.34% (120 mg) at 3 years	NA

BCG = Bacillus Calmette-Guérin; CIS = Carcinoma in situ; CRR = complete remission rate; NA = not available; OS = overall survival; PFS = progression-free survival; RFS = recurrence-free survival.

**Table 4:** Retrospective studies using BCG for CIS.

Study	Strain	Schedule	Median Follow-Up	Number of CIS Patients	CRR	RFS	PFS	OS
Chade et al. (2010) [8]	Tokyo	6 weeks + 1 reinduction (if necessary)	3.3–4.0 years	Primary CIS: 155	NA	63.2% *	55.5%	NA
Ferro et al. (2022) [44]	Unspecified	6 weeks + 3 weeks/3 years	53 months (RFS); 120 months (PFS)	Primary CIS: 172	NA	47.7%	76.2%	NA
Del Giudice et al. (2021) [45]	Connaught vs. TICE vs. RIVM	6 weeks + 3 weeks/3 years	72 months 73 months 67 months	pTis: 9; Papillary + CIS: 23 pTis: 8; Papillary + CIS: 15 pTis: 13 Papillary + CIS: 18	NA	54.1% at 5 years 61.3% at 5 years 60.2% at 5 years	74.7% at 5 years 81% at 5 years 78.2% at 5 years	NA
Hurle et al. (2019) [46]	Unspecified	6 weeks + 3 weeks/3 years	103 months	Pure CIS: 98 Non-pure CIS: 51	NA	NA	86% 71%	81% 78%
Sallami et al. (2016) [47]	Pasteur	6 weeks + 6 monthly	67.5 months	Primary CIS: 13 Concomitant CIS: 28 Secondary CIS: 6	68% (no difference between CIS groups)	84.6% at 5 years 64.3% at 5 years 50% at 5 years	87.2% at 5 years (no difference between CIS groups)	95.7% (no difference between CIS groups)
Witjes et al. (2016) [48]	Connaught vs. TICE	59% received maintenance 18% received maintenance	62.4 months	Papillary: 731; Papillary + CIS: 226 Papillary: 841; Papillary + CIS: 301	NA	54.1% 45.3%	78.8% 81.5%	73.1% 74.8%
Takashi et al. (2002) [49]	Tokyo	8 or 10 week	64.7 months	No concomitant CIS: 112 (65 received 40mg BCG, 47 received 80mg BCG) With concomitant CIS: 34 (26 received 40mg BCG, 8 received 80mg BCG)	NA	69/112 (61.6%) 18/34 (52.9%)	90% at 5 years 70% at 5 years	87% at 5 years 73% at 5 years
D’Andrea et al. (2020) [50]	TICE vs. Moreau	6-week induction 8-week induction + 6 week maintenance	41 months	Ta or T1: 311 (72 with concomitant CIS); Tis: 10 Ta or T1: 334 (16 with concomitant CIS); Tis: 5	NA	No difference	Tis subgroup favours TICE (*p* ≤ 0.01)	NA
Del Giudice et al. (2022) [51]	TICE vs. RIVM (1-to-1 propensity score matched analysis)	6 weeks + 3 weeks/3 years	53 months	No concomitant CIS: 118; Concomitant CIS: 15 No concomitant CIS: 123; Concomitant CIS: 10	NA	56% at 5 years 48% at 5 years	77% at 5 years 79% at 5 years	NA
Nowak et al. (2021) [52]	Moreau vs. TICE vs. RIVM	≥5-dose induction + ≥2-dose maintenance	40 months	No concomitant CIS: 110; Concomitant CIS: 28 No concomitant CIS: 222; Concomitant CIS: 46 No concomitant CIS: 134; Concomitant CIS: 45	NA	70.5% at 5 years 66.7% at 5 years 55.2% at 5 years	84% at 5 years 85% at 5 years 77.8% at 5 years	NA
Koguchi et al. (2020) [53]	Tokyo	6 weeks 1/2D vs. 6 weeks 1/2D + 2 weeks/3 years 1/2D	36.2 months	Ta or T1: 60; Tis: 18	NA	65.0% (induction) vs. 89.5% (maintenance) at 3 years (no difference in Ta/T1 and Tis subgroups)	97.5% (induction) vs. 97.4% (maintenance)	100% (induction) vs. 84.2% (maintenance)
Griffiths et al. (2002) [54]	Connaught	6-week induction	41 months	Primary CIS: 23 CIS + Ta: 37 (21 concomitant + 16 secondary) CIS + T1: 75 (46 concomitant + 29 secondary)	74% 70% 75%	NA	80% at 5 years 82% at 5 years 51% at 5 years	NA

BCG = Bacillus Calmette-Guérin; CIS = Carcinoma in situ; CRR = complete remission rate; NA = not available; OS = overall survival; PFS = progression-free survival; RFS = recurrence-free survival; * This percentage does not include 20 patients who developed distant metastasis and 18 who developed upper tract UC.
